# 3-Bromo-4-dibenzyl­amino-5-methoxy­furan-2(5*H*)-one

**DOI:** 10.1107/S1600536810008007

**Published:** 2010-03-06

**Authors:** Jian-Hua Fu, Zhao-Yang Li, Zhao-Yang Wang, Rui-Rong Ye

**Affiliations:** a School of Chemistry and Environment, South China Normal University, Guangzhou 510006, People’s Republic of China

## Abstract

In the the title compound, C_19_H_18_BrNO_3_, the furan­one ring is almost planar [maximum atomic deviation = 0.019 (3) Å] and is nearly perpendicular to the two phenyl rings, making dihedral angles of 88.96 (17) and 87.71 (17)°. Inter­molecular C—H⋯O hydrogen bonding is present in the crystal structure.

## Related literature

2(5*H*)-Furan­one is the simplest sub-unit of a large class of five-membered heterocyclic carbonyl compounds, see: Reva *et al.* (2008 ▶). The title compound is a derivative of 4-amino-2(5*H*)-furan­one. For the biological activity of 4-amino-2(5*H*)-furan­ones, see: Kimura *et al.* (2000 ▶); Tanoury *et al.* (2008 ▶). For the synthesis, see: Toshiyuki & Yoshikazu (1955 ▶).
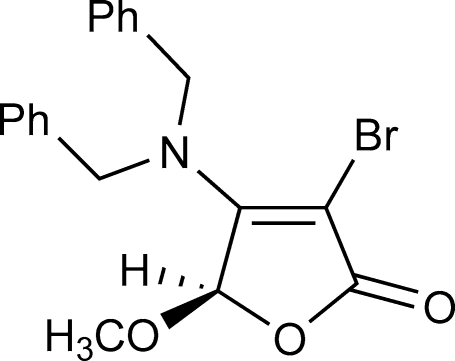


## Experimental

### 

#### Crystal data

C_19_H_18_BrNO_3_
*M* *_r_* = 388.25
Orthorhombic, 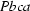

*a* = 15.756 (2) Å
*b* = 11.2475 (14) Å
*c* = 19.779 (3) Å
*V* = 3505.2 (8) Å^3^
*Z* = 8
Mo *K*α radiation
μ = 2.36 mm^−1^
*T* = 298 K
0.23 × 0.20 × 0.16 mm

#### Data collection

Bruker APEXII area-detector diffractometer
Absorption correction: multi-scan (*SADABS*; Sheldrick, 1996 ▶) *T*_min_ = 0.613, *T*_max_ = 0.704
18029 measured reflections
3429 independent reflections
2028 reflections with *I* > 2σ(*I*)
*R*_int_ = 0.054

#### Refinement

*R*[*F* ^2^ > 2σ(*F* ^2^)] = 0.036
*wR*(*F* ^2^) = 0.089
*S* = 1.00
3429 reflections
218 parameters
H-atom parameters constrained
Δρ_max_ = 0.30 e Å^−3^
Δρ_min_ = −0.27 e Å^−3^

### 

Data collection: *APEX2* (Bruker, 2008 ▶); cell refinement: *SAINT* (Bruker, 2008 ▶); data reduction: *SAINT*; program(s) used to solve structure: *SHELXS97* (Sheldrick, 2008 ▶); program(s) used to refine structure: *SHELXL97* (Sheldrick, 2008 ▶); molecular graphics: *ORTEP-3 for Windows* (Farrugia, 1997 ▶); software used to prepare material for publication: *SHELXL97*.

## Supplementary Material

Crystal structure: contains datablocks global, I. DOI: 10.1107/S1600536810008007/xu2724sup1.cif

Structure factors: contains datablocks I. DOI: 10.1107/S1600536810008007/xu2724Isup2.hkl

Additional supplementary materials:  crystallographic information; 3D view; checkCIF report

## Figures and Tables

**Table 1:** Hydrogen-bond geometry (Å, °)

*D*—H⋯*A*	*D*—H	H⋯*A*	*D*⋯*A*	*D*—H⋯*A*
C16—H16⋯O3^i^	0.98	2.49	3.396 (4)	154

Symmetry code: (i) 
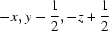
.

**Fig. 1.**
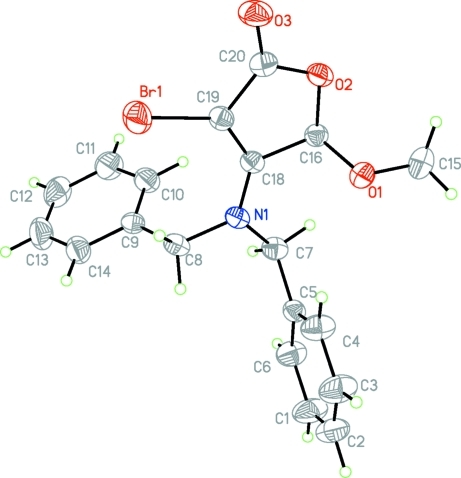
View of the title compound showing the atom-labelling scheme. Ellipsoids are drawn at the 50% probability level.

## supplementary crystallographic information

### Comment

2(5H)-Furanone is a simplest sub-unit of a large class of five membered heterocyclic carbonyl compounds (Reva *et al.*, 2008). At the same time, 4-amino-2(5H)-furanone is an attractive moiety in chemical, pharmaceutical and agrochemical research. Many 4-amino-2(5H)-furanones have been patented as prodrugs or insecticides and herbicides (Kimura *et al.*, 2000; Tanoury *et al.*, 2008).

Attracted by versatile 4-amino-2(5H)-furanones, we synthesized the title compound with 3,4-dibromo-5-methoxyfuran-2(5H)-one and dibenzylamine in the presence of potassium fluoride *via* the tandem asymmetric Michael addition-elimination reaction. With 2(5H)-furanone moiety and polyfunctional groups (carboxyl, amino, halo), the title compound is expected to be a biologically active product.

The structure of the title compound (I) is illustrated in Fig. 1. The title compound contains a five-membered furanone ring and two six-membered benzene rings. The furanone ring is approximately planar.

### Experimental

The precursor 3,4-dibromo-5-methoxyfuran-2(5H)-furanone was prepared according to the literature procedure (Toshiyuki & Yoshikazu, 1955).

After the mixture of dibenzylamine (2 mmol) and potassium fluoride (6 mmol) was dissolved in absolute tetrahydrofuran (2 ml) under nitrogen atmosphere, dichloromethane solution of 3,4-dibromo-5-methoxyfuran-2(5H)-furanone (2.0 mmol) was added. The residual liquid was dissolved in dichloromethane. The reaction was carried out under the stirring at room temperature for 48 h. Once the reaction was complete, the solvents were removed under reduced pressure. The residual solid was dissolved in dichloromethane. Then the combined organic layers from extraction were concentrated under reduced pressure, and the crude product was purified by silica gel column chromatography with the gradient mixture of petroleum ether and ethyl acetate to give the product yielding (I) 0.6224 g (80.2%).

### Refinement

H atoms were positioned in calculated positions with C—H = 0.93-0.98 Å and were refined using a riding model, with U_iso_(H) = 1.5U_eq_(C) for methyl and 1.2U_eq_(C) for the others.

### Crystal data

**Table:**

C_19_H_18_BrNO_3_	*F*(000) = 1584
*M**_r_* = 388.25	*D*_x_ = 1.471 Mg m^−^^3^
Orthorhombic, *P**b**c**a*	Mo *K*α radiation, λ = 0.71073 Å
Hall symbol: -P 2ac 2ab	Cell parameters from 2798 reflections
*a* = 15.756 (2) Å	θ = 2.5–21.2°
*b* = 11.2475 (14) Å	µ = 2.36 mm^−^^1^
*c* = 19.779 (3) Å	*T* = 298 K
*V* = 3505.2 (8) Å^3^	Block, colourless
*Z* = 8	0.23 × 0.20 × 0.16 mm

### Data collection

**Table:**

Bruker APEXII area-detector diffractometer	3429 independent reflections
Radiation source: fine-focus sealed tube	2028 reflections with *I* > 2σ(*I*)
graphite	*R*_int_ = 0.054
φ and ω scan	θ_max_ = 26.0°, θ_min_ = 2.1°
Absorption correction: multi-scan (*SADABS*; Sheldrick, 1996)	*h* = −19→18
*T*_min_ = 0.613, *T*_max_ = 0.704	*k* = −13→13
18029 measured reflections	*l* = −10→24

### Refinement

**Table:**

Refinement on *F*^2^	Primary atom site location: structure-invariant direct methods
Least-squares matrix: full	Secondary atom site location: difference Fourier map
*R*[*F*^2^ > 2σ(*F*^2^)] = 0.036	Hydrogen site location: inferred from neighbouring sites
*wR*(*F*^2^) = 0.089	H-atom parameters constrained
*S* = 1.00	*w* = 1/[σ^2^(*F*_o_^2^) + (0.0297*P*)^2^ + 1.9449*P*] where *P* = (*F*_o_^2^ + 2*F*_c_^2^)/3
3429 reflections	(Δ/σ)_max_ = 0.002
218 parameters	Δρ_max_ = 0.30 e Å^−^^3^
0 restraints	Δρ_min_ = −0.27 e Å^−^^3^

### Special details

**Table:**

Experimental. ^1^H NMR (400 MHz, CDCl_3_, TMS): 3.52 (3H, *s*, CH, CH_3_), 4.53 (2H, *d*, CH, CH_2_), 4.90 (2H, *d*, CH, CH_2_), 5.74 (1H, *s*, CH), 7.21-7.42 (10H, *m*, CH, Ar-H);
Geometry. All esds (except the esd in the dihedral angle between two l.s. planes) are estimated using the full covariance matrix. The cell esds are taken into account individually in the estimation of esds in distances, angles and torsion angles; correlations between esds in cell parameters are only used when they are defined by crystal symmetry. An approximate (isotropic) treatment of cell esds is used for estimating esds involving l.s. planes.
Refinement. Refinement of F^2^ against ALL reflections. The weighted R-factor wR and goodness of fit S are based on F^2^, conventional R-factors R are based on F, with F set to zero for negative F^2^. The threshold expression of F^2^ > 2sigma(F^2^) is used only for calculating R-factors(gt) etc. and is not relevant to the choice of reflections for refinement. R-factors based on F^2^ are statistically about twice as large as those based on F, and R- factors based on ALL data will be even larger.

### Fractional atomic coordinates and isotropic or equivalent isotropic displacement parameters (Å^2^)

**Table:**

	*x*	*y*	*z*	*U*_iso_*/*U*_eq_	
Br1	0.09981 (2)	1.14889 (3)	0.35209 (2)	0.07708 (16)	
C9	0.24254 (19)	0.8956 (3)	0.36299 (14)	0.0447 (7)	
C5	0.29710 (17)	0.8590 (3)	0.16073 (14)	0.0440 (7)	
C8	0.25104 (19)	0.9658 (3)	0.29832 (14)	0.0506 (8)	
H8A	0.2423	1.0493	0.3083	0.061*	
H8B	0.3085	0.9570	0.2815	0.061*	
C6	0.3624 (2)	0.7779 (3)	0.15594 (17)	0.0689 (10)	
H6	0.3598	0.7075	0.1805	0.083*	
C7	0.22153 (18)	0.8294 (2)	0.20442 (15)	0.0495 (8)	
H7A	0.1754	0.8027	0.1757	0.059*	
H7B	0.2365	0.7644	0.2344	0.059*	
C10	0.1799 (2)	0.8127 (3)	0.37322 (17)	0.0618 (9)	
H10	0.1395	0.7997	0.3398	0.074*	
C4	0.3031 (2)	0.9608 (3)	0.12329 (18)	0.0674 (10)	
H4	0.2598	1.0168	0.1252	0.081*	
C14	0.3011 (2)	0.9133 (3)	0.41324 (17)	0.0625 (9)	
H14	0.3436	0.9696	0.4072	0.075*	
C11	0.1764 (2)	0.7486 (3)	0.43241 (19)	0.0750 (11)	
H11	0.1335	0.6928	0.4388	0.090*	
C13	0.2981 (3)	0.8493 (4)	0.47231 (18)	0.0828 (12)	
H13	0.3385	0.8623	0.5058	0.099*	
C12	0.2358 (3)	0.7666 (4)	0.48199 (18)	0.0806 (11)	
H12	0.2337	0.7230	0.5219	0.097*	
C2	0.4371 (3)	0.9017 (4)	0.0793 (2)	0.0818 (12)	
H2	0.4844	0.9168	0.0525	0.098*	
C1	0.4314 (2)	0.7997 (4)	0.1153 (2)	0.0858 (12)	
H1	0.4747	0.7438	0.1125	0.103*	
C3	0.3730 (3)	0.9814 (4)	0.0826 (2)	0.0840 (12)	
H3	0.3760	1.0509	0.0573	0.101*	
N1	0.19202 (15)	0.9304 (2)	0.24524 (12)	0.0448 (6)	
O1	0.08872 (13)	0.95812 (19)	0.12416 (10)	0.0558 (6)	
O3	−0.06901 (14)	1.1434 (2)	0.26344 (12)	0.0691 (6)	
O2	−0.02363 (12)	0.99727 (18)	0.19541 (10)	0.0532 (5)	
C16	0.05326 (18)	0.9313 (3)	0.18643 (14)	0.0434 (7)	
H16	0.0426	0.8457	0.1904	0.052*	
C18	0.11283 (18)	0.9735 (2)	0.24190 (14)	0.0400 (7)	
C20	−0.0134 (2)	1.0746 (3)	0.24854 (16)	0.0497 (8)	
C19	0.06917 (18)	1.0562 (2)	0.27812 (14)	0.0446 (7)	
C15	0.0499 (3)	0.8984 (4)	0.07008 (18)	0.0898 (13)	
H15A	0.0518	0.8142	0.0780	0.135*	
H15B	0.0794	0.9166	0.0289	0.135*	
H15C	−0.0082	0.9235	0.0663	0.135*	

### Atomic displacement parameters (Å^2^)

**Table:**

	*U*^11^	*U*^22^	*U*^33^	*U*^12^	*U*^13^	*U*^23^
Br1	0.0724 (3)	0.0756 (3)	0.0833 (3)	0.0017 (2)	−0.0117 (2)	−0.0326 (2)
C9	0.0377 (17)	0.0467 (16)	0.0498 (19)	0.0101 (14)	−0.0009 (15)	−0.0007 (14)
C5	0.0398 (17)	0.0478 (17)	0.0445 (18)	0.0011 (15)	−0.0038 (13)	0.0051 (15)
C8	0.0364 (17)	0.0554 (19)	0.060 (2)	−0.0035 (15)	−0.0035 (15)	0.0059 (16)
C6	0.059 (2)	0.074 (2)	0.073 (2)	0.0192 (19)	0.0072 (19)	0.026 (2)
C7	0.0452 (18)	0.0435 (18)	0.060 (2)	0.0056 (14)	0.0007 (15)	0.0084 (15)
C10	0.053 (2)	0.075 (2)	0.057 (2)	−0.0080 (18)	−0.0030 (16)	0.0139 (18)
C4	0.060 (2)	0.057 (2)	0.085 (3)	0.0059 (17)	0.0155 (19)	0.020 (2)
C14	0.061 (2)	0.068 (2)	0.059 (2)	0.0024 (18)	−0.0100 (18)	−0.0032 (19)
C11	0.079 (3)	0.081 (3)	0.066 (2)	−0.006 (2)	0.005 (2)	0.017 (2)
C13	0.088 (3)	0.102 (3)	0.058 (3)	0.008 (3)	−0.022 (2)	−0.005 (2)
C12	0.097 (3)	0.091 (3)	0.054 (2)	0.015 (3)	0.002 (2)	0.019 (2)
C2	0.059 (2)	0.103 (3)	0.083 (3)	−0.005 (2)	0.023 (2)	0.006 (3)
C1	0.060 (2)	0.102 (3)	0.095 (3)	0.026 (2)	0.024 (2)	0.017 (3)
C3	0.080 (3)	0.075 (3)	0.098 (3)	−0.007 (2)	0.026 (2)	0.030 (2)
N1	0.0362 (14)	0.0476 (14)	0.0506 (15)	−0.0009 (12)	−0.0020 (11)	0.0026 (12)
O1	0.0549 (13)	0.0668 (14)	0.0457 (13)	−0.0002 (11)	0.0038 (11)	0.0049 (11)
O3	0.0516 (13)	0.0647 (14)	0.0909 (18)	0.0162 (12)	0.0008 (12)	−0.0133 (13)
O2	0.0393 (12)	0.0610 (13)	0.0594 (14)	0.0064 (10)	−0.0041 (10)	−0.0071 (11)
C16	0.0411 (17)	0.0447 (17)	0.0442 (18)	−0.0011 (14)	0.0014 (14)	0.0013 (14)
C18	0.0381 (17)	0.0366 (16)	0.0452 (17)	−0.0082 (13)	0.0016 (13)	0.0085 (13)
C20	0.048 (2)	0.0440 (18)	0.057 (2)	−0.0009 (16)	0.0058 (16)	0.0004 (16)
C19	0.0427 (17)	0.0406 (16)	0.0506 (18)	−0.0044 (14)	−0.0017 (14)	−0.0012 (14)
C15	0.086 (3)	0.133 (4)	0.050 (2)	−0.001 (3)	0.004 (2)	−0.015 (2)

### Geometric parameters (Å, °)

**Table:**

Br1—C19	1.860 (3)	C11—H11	0.9300
C9—C14	1.371 (4)	C13—C12	1.366 (5)
C9—C10	1.372 (4)	C13—H13	0.9300
C9—C8	1.509 (4)	C12—H12	0.9300
C5—C4	1.367 (4)	C2—C3	1.351 (5)
C5—C6	1.378 (4)	C2—C1	1.353 (5)
C5—C7	1.508 (4)	C2—H2	0.9300
C8—N1	1.458 (3)	C1—H1	0.9300
C8—H8A	0.9700	C3—H3	0.9300
C8—H8B	0.9700	N1—C18	1.340 (3)
C6—C1	1.374 (5)	O1—C16	1.386 (3)
C6—H6	0.9300	O1—C15	1.404 (4)
C7—N1	1.469 (3)	O3—C20	1.206 (3)
C7—H7A	0.9700	O2—C20	1.374 (3)
C7—H7B	0.9700	O2—C16	1.432 (3)
C10—C11	1.376 (4)	C16—C18	1.520 (4)
C10—H10	0.9300	C16—H16	0.9800
C4—C3	1.383 (5)	C18—C19	1.361 (4)
C4—H4	0.9300	C20—C19	1.441 (4)
C14—C13	1.373 (5)	C15—H15A	0.9600
C14—H14	0.9300	C15—H15B	0.9600
C11—C12	1.371 (5)	C15—H15C	0.9600
			
C14—C9—C10	118.4 (3)	C13—C12—H12	120.3
C14—C9—C8	118.6 (3)	C11—C12—H12	120.3
C10—C9—C8	123.0 (3)	C3—C2—C1	119.1 (4)
C4—C5—C6	117.7 (3)	C3—C2—H2	120.4
C4—C5—C7	123.4 (3)	C1—C2—H2	120.4
C6—C5—C7	118.9 (3)	C2—C1—C6	120.8 (4)
N1—C8—C9	114.3 (2)	C2—C1—H1	119.6
N1—C8—H8A	108.7	C6—C1—H1	119.6
C9—C8—H8A	108.7	C2—C3—C4	120.8 (4)
N1—C8—H8B	108.7	C2—C3—H3	119.6
C9—C8—H8B	108.7	C4—C3—H3	119.6
H8A—C8—H8B	107.6	C18—N1—C8	122.1 (2)
C1—C6—C5	120.8 (3)	C18—N1—C7	123.2 (2)
C1—C6—H6	119.6	C8—N1—C7	113.9 (2)
C5—C6—H6	119.6	C16—O1—C15	113.4 (2)
N1—C7—C5	113.2 (2)	C20—O2—C16	108.9 (2)
N1—C7—H7A	108.9	O1—C16—O2	109.8 (2)
C5—C7—H7A	108.9	O1—C16—C18	108.9 (2)
N1—C7—H7B	108.9	O2—C16—C18	105.7 (2)
C5—C7—H7B	108.9	O1—C16—H16	110.7
H7A—C7—H7B	107.7	O2—C16—H16	110.7
C9—C10—C11	120.7 (3)	C18—C16—H16	110.7
C9—C10—H10	119.6	N1—C18—C19	133.8 (3)
C11—C10—H10	119.6	N1—C18—C16	119.9 (2)
C5—C4—C3	120.7 (3)	C19—C18—C16	106.3 (2)
C5—C4—H4	119.7	O3—C20—O2	120.5 (3)
C3—C4—H4	119.7	O3—C20—C19	130.5 (3)
C9—C14—C13	121.2 (3)	O2—C20—C19	109.0 (3)
C9—C14—H14	119.4	C18—C19—C20	109.9 (3)
C13—C14—H14	119.4	C18—C19—Br1	131.8 (2)
C12—C11—C10	120.2 (4)	C20—C19—Br1	118.2 (2)
C12—C11—H11	119.9	O1—C15—H15A	109.5
C10—C11—H11	119.9	O1—C15—H15B	109.5
C12—C13—C14	120.0 (4)	H15A—C15—H15B	109.5
C12—C13—H13	120.0	O1—C15—H15C	109.5
C14—C13—H13	120.0	H15A—C15—H15C	109.5
C13—C12—C11	119.4 (4)	H15B—C15—H15C	109.5

### Hydrogen-bond geometry (Å, °)

**Table:**

*D*—H···*A*	*D*—H	H···*A*	*D*···*A*	*D*—H···*A*
C16—H16···O3^i^	0.98	2.49	3.396 (4)	154

Symmetry codes: (i) −*x*, *y*−1/2, −*z*+1/2.

## References

[bb1] Bruker (2008). *APEX2* and *SAINT* Bruker AXS Inc., Madison, Wisconsin, USA.

[bb2] Farrugia, L. J. (1997). *J. Appl. Cryst.* **30**, 565.

[bb3] Kimura, Y., Mizuno, T., Kawano, T., Okada, K. & Shimad, A. (2000). *Phytochemistry*, **53**, 829–831. 10.1016/s0031-9422(99)00492-6 10820786

[bb8] Reva, I., Nowak, M. J., Lapinski, L. & Fausto, R. (2008). *Chem. Phys. Lett.* **452**, 20–28.

[bb4] Sheldrick, G. M. (1996). *SADABS* University of Göttingen, Germany.

[bb5] Sheldrick, G. M. (2008). *Acta Cryst.* A**64**, 112–122. 10.1107/S0108767307043930 18156677

[bb6] Tanoury, G. J., Chen, M. Z., Dong, Y., Forslund, R. E. & Magdziak, D. (2008). *Org. Lett.* **10**, 185–188. 10.1021/ol702532h 18081302

[bb7] Toshiyuki, S. & Yoshikazu, H. (1955). *Nippon Kagaku Kaishi*, **58**, 692–693.

